# Non-Pharmaceutical Interventions Implemented to Control the COVID-19 Were Associated With Reduction of Influenza Incidence

**DOI:** 10.3389/fpubh.2022.773271

**Published:** 2022-02-18

**Authors:** Qing-Mei Huang, Wei-Qi Song, Fen Liang, Bi-Li Ye, Zhi-Hao Li, Xi-Ru Zhang, Wen-Fang Zhong, Pei-Dong Zhang, Dan Liu, Dong Shen, Pei-Liang Chen, Qu Liu, Xingfen Yang, Chen Mao

**Affiliations:** ^1^Department of Epidemiology, School of Public Health, Southern Medical University, Guangzhou, China; ^2^Longgang Center for Disease Control and Prevention in Shenzhen, Shenzhen, China; ^3^Food Safety and Health Research Center, School of Public Health, Southern Medical University, Guangzhou, China

**Keywords:** influenza, non-pharmaceutical intervention, disease surveillance, COVID-19, pandemic

## Abstract

**Background:**

Non-pharmaceutical interventions were implemented in most countries to reduce the transmission of COVID-19. We aimed to describe the incidence of influenza in four countries in the 2019–2020 season and examined the effect of these non-pharmaceutical interventions on the incidence of influenza.

**Methods:**

We used the network surveillance data from 2015 to 2020 to estimate the percentage increase in influenza cases to explore the effect of non-pharmaceutical interventions implemented to control the COVID-19 on the incidence of influenza in China, the United States, Japan, and Singapore.

**Results:**

We found that the incidence of influenza has been almost zero and reached a persistent near-zero level for a continuous period of six months since epidemiologic week 14 of 2020 in the four countries. Influenza incidence decreased by 77.71% and 60.50% in the early days of COVID-19 in the 2019–2020 season compared to the same period in preceding years in Japan and Singapore, respectively. Furthermore, influenza incidence decreased by 60.50–99.48% during the period of compulsory interventions in the 2019–2020 season compared to the same period in preceding years in the four countries.

**Conclusion:**

These findings suggest that the application of non-pharmaceutical interventions, even everyday preventive action, was associated with a reduction of influenza incidence, which highlights that more traditional public health interventions need to be reasserted and universalized to reduce influenza incidence.

## Introduction

The Coronavirus Disease 2019 (COVID-19) was first reported in Wuhan, China on 31 December 2019, and spread quickly across the world after January 2020 (1). The World Health Organization (WHO) has declared it a global pandemic on 11 March (2). Faced with a devastating pandemic, many nations and regions subsequently implemented incremental public health interventions to mitigate COVID-19 transmission, which comprised travel restrictions, border closures, respiratory etiquette, hand hygiene, disinfection, wearing a face mask, closing school, and public gathering cancellations (1, 3–6).

Prior to the COVID-19 pandemic, the annual incidence of respiratory illness was mostly comprised of influenza. Influenza is a significant public health problem, accounting for about 291,000 to 646,000 seasonal influenza-associated respiratory deaths annually (7). COVID-19 and influenza may be closely interconnected. Influenza shares similar modes of transmission through droplet and contact routes with COVID-19 (8). Similar to COVID-19, influenza outbreaks tend to occur in people-intensive places, such as schools and childcare facilities. Therefore, one question emerges: whether non-pharmaceutical interventions implemented to control the spread of COVID-19 were associated with the reduced incidence of influenza.

Most policymakers agree that vaccination is the most effective method for the prevention and control of infection (9). Several preventive actions that are aimed at limiting the spread of the disease were frequently forgotten or ignored. However, a growing body of research suggests that non-pharmaceutical interventions might play a salubrious role in delaying the temporal effect of a pandemic; reducing the overall and peak incidence (10–12). Such measures could potentially alleviate the uneven distribution of vaccines in various regions of the world and decrease the burden on health care services (13, 14).

In this study, we used the network surveillance data to explore the effect of non-pharmaceutical interventions implemented to control the spread of COVID-19 on the incidence of influenza.

## Methods

### Data Collection

In this observational study, we estimated the influenza data based on the publicly available sentinel surveillance systems of influenza, influenza-like illness (ILI), or acute respiratory infection (ARI) cases in sentinel clinics/ hospitals from October 2015 to September 2020 in China, the United States, Japan, and Singapore. The number of influenza cases in China and Japan was respectively obtained from the National Health Commission of the People's Republic of China (http://www.nhc.gov.cn/) and the National Institute of Infectious Disease (NIID) (https://www.niid.go.jp/niid/en/idwr-e.html). The weekly number of ILI and the weekly proportion of specimens positive for influenza in the United States were obtained from the World Health Organization (WHO) global influenza epidemiological data sharing platform (FluID) (https://www.who.int/influenza/surveillance_monitoring/fluid/en/). The weekly number of ARI, the proportion of ILI among the polyclinic attendances for ARI, and the weekly proportion of specimens positive for influenza in Singapore were obtained from the Ministry of Health of Singapore (https://www.moh.gov.sg/). At present, the WHO runs the largest human influenza surveillance network, which includes 152 institutions from 114 countries (15). While the Flunet accommodates both qualitative and quantitative data which facilitates the tracking of global trends, spread, intensity, and impact of influenza (16), local and national prospective influenza and influenza-like illness surveillance systems also provide important information to policymakers and public health practitioners for situational awareness during periods of influenza activity. The surveillance systems rely on both virological and clinical data, including number of ILI and proportion of specimens positive for influenza. Furthermore, those surveillance networks publish regional data from surveillance systems on a monthly or weekly basis.

In addition, we captured all the available public health documents on non-pharmaceutical interventions for controlling COVID-19 implemented by these four countries during the COVID-19 epidemic, including staff reporting, the corpus of published historical, medical, and public health literature on the COVID-19 epidemic, government websites and communications, media statements, and fresh news (sources is available in the Supplementary Material).

### Interventions Definitions

Two overarching interventions, including compulsory interventions and routine prevention interventions, have been used in the four countries. The compulsory non-pharmaceutical interventions included visa restrictions, face mask ordinances, public gathering bans (including closure of saloons, public entertainment venues, churches, restaurants, churches, and dance halls, and suspension of sporting events and parades), closure of school, and work from home. Specifically, these non-pharmaceutical interventions were legally enforced and affected large segments of the country's population. The routine prevention interventions are also known as everyday preventive actions. The WHACK acronym created by the City of Berkley Division of Public Health is widely used to name routine prevention interventions, including washing or sanitizing your hands often, staying at home when you are sick, avoiding touching your eyes, nose, and mouth, covering your coughs and sneezes, and keeping away from sick people.

Based on an estimated a median incubation period of 1–2 days (17), we estimated that the association of non-pharmaceutical interventions with the incidence rate of influenza occurred in their actual month/week of implementation.

### Classification of Three Time Periods

To better reflect the dynamics of the influenza epidemic and corresponding interventions of COVID-19, three periods were classified based on important dates that could affect the influenza virus transmission in four countries (Figure 2). The time before the date of the reported COVID-19 first case was considered as the first period when no COVID-19-specific interventions were imposed. The second period was the early days of COVID-19, which was the time between the date of the first reported COVID-19 case and the date of implementing the first compulsory non-pharmaceutical intervention. No strong intervention was imposed in the early of COVID-19. During this period, the governments have mostly advised the routine prevention interventions, such as respiratory etiquette, hand hygiene, and disinfection, and citizen support and willingness have been essential. The third period was the time after the date of implementing the first compulsory non-pharmaceutical interventions when the governments announced and implemented the most compulsory non-pharmaceutical interventions such as visa restrictions, face mask ordinances, public gathering bans, closure of school, and closure of workplace. On the whole, the 2019–2020 influenza season was divided into three periods: no intervention prior to COVID-19, routine prevention interventions in the early days of COVID-19, and the period of compulsory interventions (details in the Supplementary Material).

### Data Analysis

We estimated weekly the number of influenza cases by multiplying the weekly number of ILI by the weekly proportion of specimens positive for influenza in the United States and Singapore. Furthermore, the incidence of influenza of the four countries is equal to the number of influenza cases per month/week divided by the total population at the end of the previous year, which are better correlate of the incidence of influenza virus infections than either ILI rates or laboratory detection rates alone (18).

To explore the association of COVID-19 interventions with influenza incidence, we estimated the percentage increase in influenza cases to compare the observed incidence of influenza in the three segments with average from corresponding periods in the four preceding epidemiologic years (from 2015–2016 season to the 2018–2019 season). The 2.5th and 97.5th percentile of each set of the 10,000 estimates yielded a 95% uncertainty interval (UI).

## Results

### Difference of Influenza Between the 2019 and 2020 Season and the Four Preceding Epidemiologic Years

**Figure 1:**
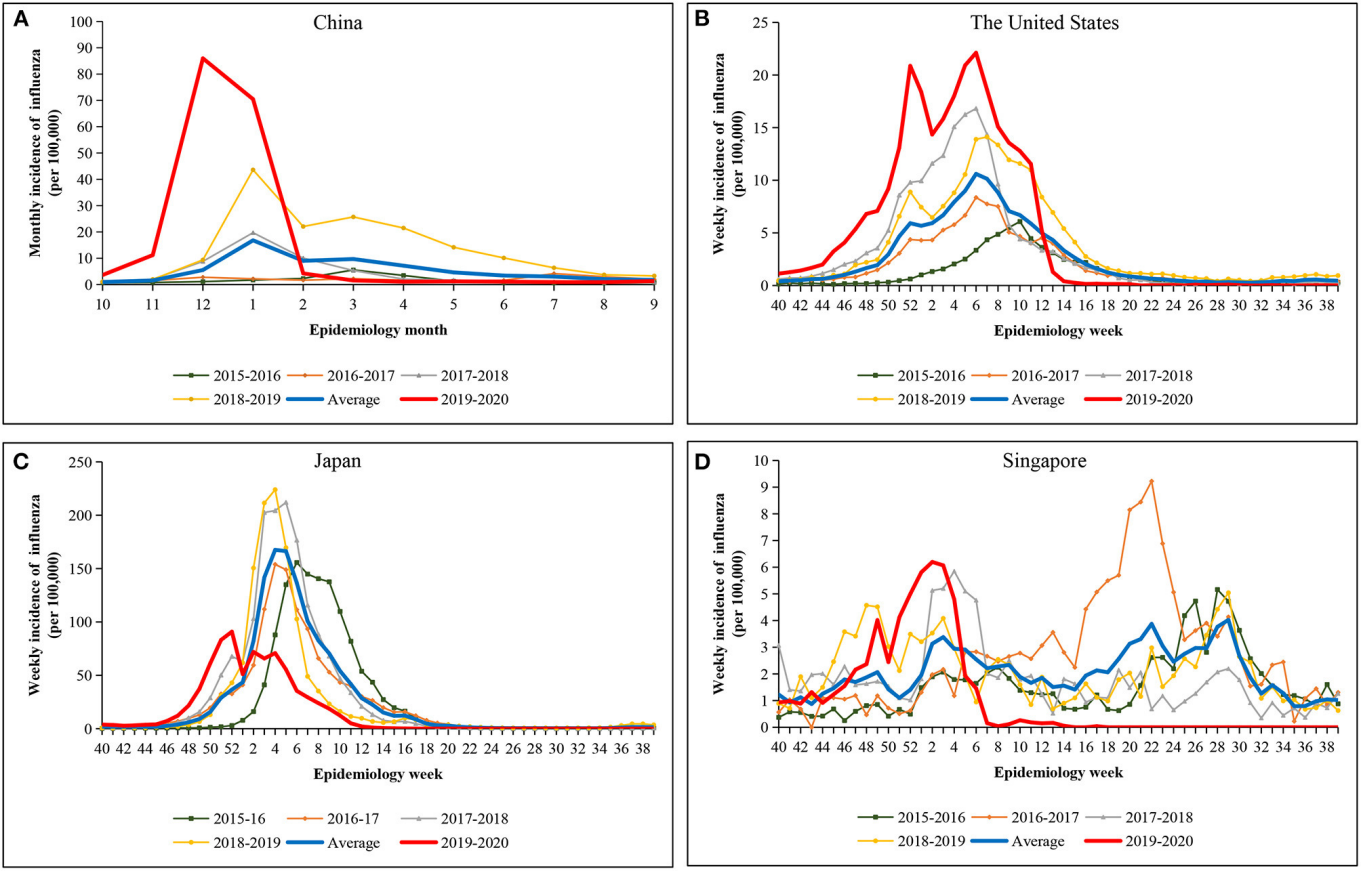
Non-pharmaceutical interventions across the four countries during the COVID-19 outbreak, and the observed (red line) and average (blue line) epidemic curve of influenza. **(A)** China. **(B)** The United States. **(C)** Japan. **(D)** Singapore.

We compared indicators of influenza transmissibility in the 2019–2020 season against the average from corresponding periods in the four preceding epidemiologic years (Figure 1). The influenza epidemic peaked in epidemiologic December 2020 in China, while the influenza epidemic reached its double peak in epidemiologic week 52 of 2019 and epidemiologic week 6 of 2020 in the United States. The peaks of influenza incidence in the 2019–2020 season were higher than those in previous years in China and the United States. However, influenza incidence declined to below the average of the preceding years by February 2020 and epidemiologic week 13 of 2020 in China and the United States, respectively. In Japan, the influenza epidemic peaked in epidemiologic week 52 of 2019 in Japan, but declined to below the average of the preceding years by epidemiologic week 2. In Singapore, the influenza epidemic peaked in epidemiologic week 2 of 2020, but declined to below the average of the preceding years by epidemiologic week 5. Interestingly, since epidemiologic week 14 of 2020, the incidence of influenza has been almost zero and reached a persistent near-zero level for a continuous period of six months in the four countries.

### Association of COVID-19 Interventions With Incidence of Influenza

**Figure 2:**
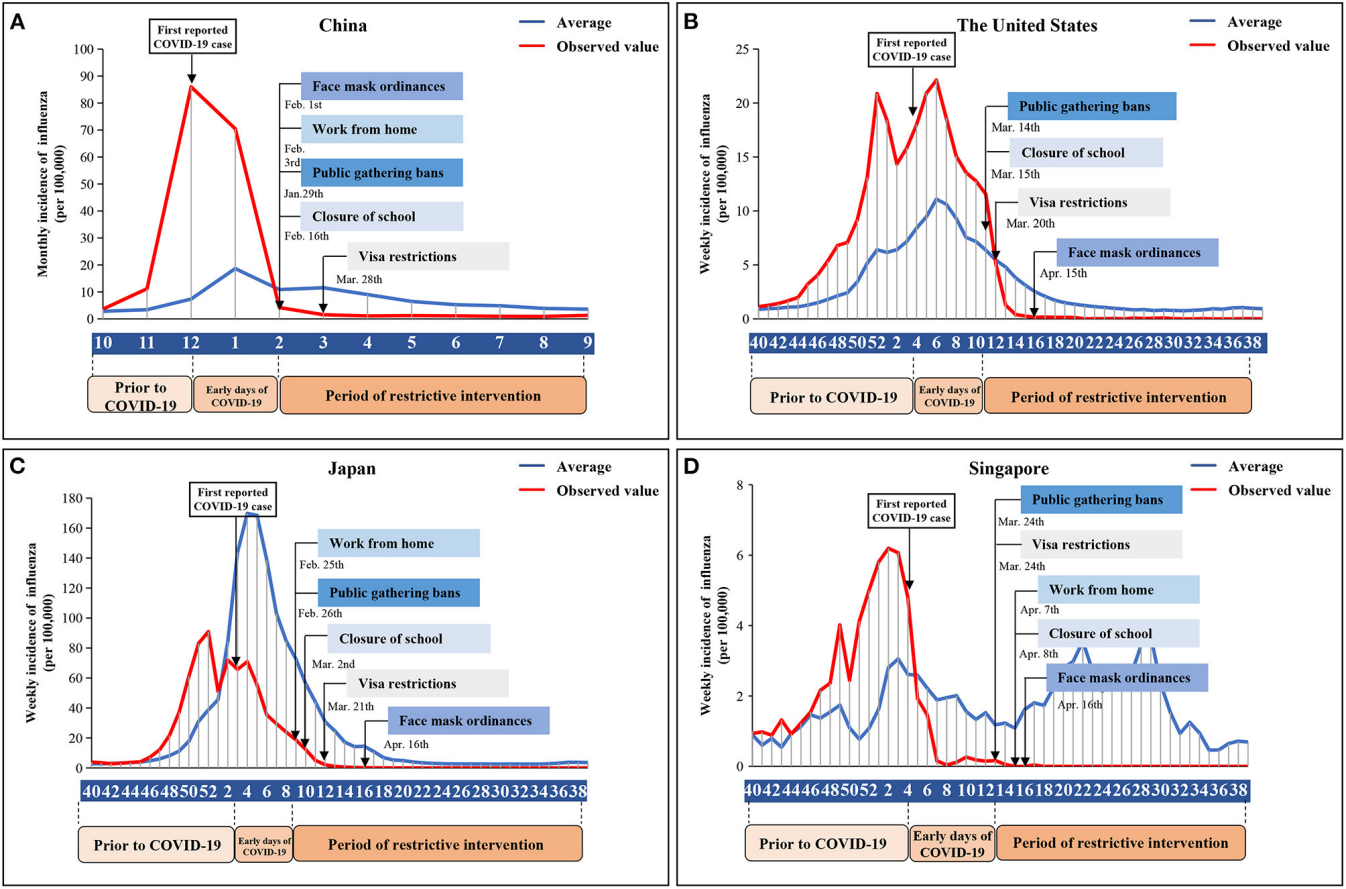
Incidence of influenza during the 2019–2020 season (red line) compared with the average from corresponding periods in the four preceding epidemiologic years (blue line). **(A)** China. **(B)** The United States. **(C)** Japan. **(D)** Singapore.

Figure 2 shows the timeline of COVID-19 and compulsory non-pharmaceutical interventions that were implemented by the governments in the four countries, including visa restrictions, face mask ordinances, work from home, public gathering bans, and closure of school. In China and the United States, the incidence of influenza significantly declined following the first compulsory interventions of COVID-19. The incidence of influenza in February decreased by 94.00% compared to January in China, while the incidence of influenza in epidemiologic week 16 decreased by 98.70% compared to epidemiologic week 1 in the United States (Figures 2A,B). In Japan and Singapore, the weekly incidence of influenza significantly declined following the first case of COVID-19. The incidence of influenza in the epidemiologic week 8 decreased by 63.64% compared to the epidemiologic week 3 of 2020 in Japan, and the incidence of influenza in epidemiologic week 12 decreased by 96.88% compared to the epidemiologic week 4 of 2020 in Singapore (Figures 2C,D).

Compared to the average from corresponding periods in the four preceding epidemiologic years, the increase in the observed number of influenza cases in the 2019–2020 season in four countries changed over time in three periods (Table 1; Figure 2). In China and the United States, influenza cases exploding prior to the COVID-19 and in the early days of the COVID-19. However, influenza incidence decreased by 77.71 and 60.50% in the period of restrictive intervention compared with the average. In Japan and Singapore, it is estimated that the observed incidence of influenza might have been 65.26 and 48.62% lower than the average in the early days of COVID-19 and the reducing range increased (88.70 and 99.48%) during the period of compulsory interventions.

**Table 1:** Incidence of influenza in the 2019–2020 season stratified by time periods and average in four countries.

**Time Periods**	**Average^a^**		**Observed Value**	
	**No. of influenza cases**	**Incidence** **per 100,000** **(95% UI)**	**No. of influenza cases**	**Incidence** **per 100,000** **(95% UI)**
China				
Prior to the COVID-19	87,261	6.23	206,870	14.83
Early days of the COVID-19	3,64,053	26.05 (25.96–26.13)	2,186,314	74.69
The period of compulsory interventions	7,78,273	55.59 (51073–16.95)	173,531	12.39 (10.65–14.42)
The United States				
Prior to the COVID-19	1,60,510	48.88 (48.64–49.12)	412,832	125.72 (125.33–126.10)
Early days of the COVID-19	2,09,294	63.65 (63.38–63.93)	398,143	121.09 (120.71–121.47)
The period of compulsory interventions	1,65,566	50.35 (50.11–50.60)	65,414	19.89 (19.74–20.05)
Japan				
Prior to the COVID-19	3,38,063	267.46 (266.56–268.36)	578,681	457.83 (456.65–459.01)
Early days of the COVID-19	10,21,127	808.72 (807.16–810.28)	354,676	280.90 (279.98–281.82)
The period of compulsory interventions	4,53,393	359.08 (358.04–360.13)	51,232	40.58 (40.22–40.93)
Singapore				
Prior to the COVID-19	1,215	21.42 (20.24–22.66)	2,605	45.94 (44.19–47.73)
Early days of the COVID-19	1,009	17.69 (16.62–18.82)	518	9.09 (8.32–9.90)
The period of compulsory interventions	2,848	49.93 (48.12–51.80)	15	0.26 (0.15–0.43)

a *The average annual number or average annual incidence of influenza cases from the 2015–2016 season to 2018–2019 season*.

## Discussion

In this study, we found that the incidence of influenza has been almost zero and reached a persistent near-zero level for a continuous period of six months since epidemiologic week 14 of 2020 in China, the United States, Japan, and Singapore. Furthermore, the incidence of influenza decreased by 60.50–99.48% during the period of compulsory interventions compared to the average from corresponding periods in the four preceding epidemiologic years.

The present study provides further support that the incidence rates of influenza in the 2019–2020 season were lower compared to the preceding years, in line with previous studies in France, Hong Kong, Thailand, and Taiwan (19–22). However, most of the previous studies used ILI incidence to be the proxy indicator of influenza incidence. It is likely that there is a lack of correlation between influenza trends and ILI trends during the COVID-19 outbreak. ILI patients might be COVID-19 cases instead of influenza cases due to similar clinical symptoms (23). Compared with the previous studies, a more accurate indicator was used in the present study. We estimated the number of influenza cases per week by multiplying the number of influenza-like illnesses per week by proportions of specimens positive for influenza, which is a better correlate of the incidence of influenza virus infections than either influenza-like illness rates or laboratory detection rates alone.

The present study also showed that the reduction in transmissibility of influenza might be associated with the outbreak of COVID-19 and the package of non-pharmaceutical interventions implemented to control the spread of COVID-19. There are common clinical symptoms, such as fever and cough, between COVID-19 and influenza (24). Individuals with those clinical symptoms might be warned to be more likely to maintain social distancing and wear a mask to reduce the risk of transmitting the virus. In addition, influenza shares similar modes of transmission through respiratory and contact routes with COVID-19 (8). Containment and mitigation measures for COVID-19 might make it difficult to transmit the influenza virus. Furthermore, travel restrictions and border closures may result in a potential reduction in the importation of influenza cases, which might reduce the incidence of influenza (25).

In addition, we found that the observed incidence of influenza increased significantly in the early days of COVID-19 in China and the United States, compared to the average. This may be because patients with influenza were more likely to see a doctor for fear of being diagnosed with COVID-19. However, it is estimated that the incidence of influenza cases might have been lower than the average because of the routine prevention interventions in Japan and Singapore. Since Japan and Singapore are geographically proximal to China, the impact of the COVID-19 outbreak in China might have affected the government and public more directly in these countries. Another explanation is that mask-wearing may be more common in East Asia than in the US after the first reported COVID-19 case, which may be due to the inherent national culture (26–28).

We found that the compulsory non-pharmaceutical interventions coincided with a substantial reduction in influenza transmission in all four countries. Previous evidence showed that the reduction in transmission of influenza was associated with school closures in Hong Kong (29). Another study in the United States also showed that the combination of school closure and public gathering bans was significantly associated with reductions in the weekly excess death rate of influenza (10). Furthermore, the decrease in influenza incidence was greater during the period of restrictive intervention than during the early days of COVID-19. It's worth noting that the incidence rates of influenza were not only lower but also reached a persistent near-zero level for a continuous period of six months. The reason may be that the compulsory non-pharmaceutical interventions focused on legal enforcement and affected large segments of the country's population, while routine prevention interventions focused on the voluntary self-protection of the public.

An influenza vaccine response is almost certain to be a global necessity in the influenza epidemic response, immunizing the population to stop virus importation and transmission (30). However, uncertainties exist about immunogenicity (31), effectiveness (32, 33), and timing of influenza vaccine production. In addition, inequities in immunization programs and vaccine hesitancy are common in all countries and regions (34–36). As an unplanned vaccine, the influenza vaccine has a very low coverage rate in economically underdeveloped areas, especially in poor areas. Vaccination coverage varied by country. For example, coverage was 45.3% among adults ≥ 18 years in 2018–2019 in the United States (37), while coverage for influenza vaccine was ~2% in 2018–2019 in China (38). Non-pharmaceutical interventions might play a salubrious role in counteracting the negative effects of the reduction of vaccine effectiveness and delaying vaccine production (13, 14), and be an important adjunct to influenza vaccination programs to reduce the number of influenzas (39). Maintenance of everyday preventive actions for controlling the coming winter influenza is crucial. In the case of influenza pandemic and delays to mass vaccination, public health officials could announce and implement the most compulsory non-pharmaceutical interventions such as visa restrictions, face mask ordinances, closure of school, public gathering bans, and closure of workplace.

Our study has some limitations. First, this study was an ecological study, precluding causal inference. Second, data were extracted from the infectious disease surveillance system, and the incidence of influenza might be affected by the number of influenza tests during the COVID-19 epidemic. However, the incidence of influenza was a better correlate of the incidence of influenza virus infections than either influenza-like illness rates or laboratory detection rates alone. Finally, the measures did not include information about climate factors. Seasonal trends and potential climate changes may also have an impact on the incidence of influenza.

## Conclusion

In this observational study, there was a marked decline in the incidence of influenza after the implementation of non-pharmaceutical interventions for COVID-19 in China, the United States, Japan, and Singapore, and likely so in the foreseeable future if these behaviors become normalized. Influenza should continue, and more traditional public health interventions need to be reasserted and universalized.

## Data Availability Statement

The datasets presented in this study can be found in online repositories. The names of the repository/repositories and accession number(s) can be found in the article/Supplementary Material.

## Author Contributions

Q-MH, W-QS, and FL contributed to the statistical analyses and the manuscript. CM, XY, and QL directed the study. B-LY, Z-HL, X-RZ, W-FZ, P-DZ, DL, DS, and P-LC contributed to the data cleaning. All authors critically reviewed the manuscript for important intellectual content. CM is the study guarantor. The corresponding author attests that all listed authors meet authorship criteria and that no others meeting the criteria have been omitted. All authors approved the final version of the manuscript.

## Funding

This work was supported by the Project Supported by Guangdong Province Universities and Colleges Pearl River Scholar Funded Scheme (2019), the Chang Jiang Scholars Program (2020), and the Construction of High-level University of Guangdong (G621331128).

## Conflict of Interest

The authors declare that the research was conducted in the absence of any commercial or financial relationships that could be construed as a potential conflict of interest.

## Publisher's Note

All claims expressed in this article are solely those of the authors and do not necessarily represent those of their affiliated organizations, or those of the publisher, the editors and the reviewers. Any product that may be evaluated in this article, or claim that may be made by its manufacturer, is not guaranteed or endorsed by the publisher.
