# Supplementary material for: Non-Pharmaceutical Interventions Implemented to Control the COVID-19 Were Associated With Reduction of Influenza Incidence
Source: Front Public Health. 2022 Feb 18;10:773271. doi: 10.3389/fpubh.2022.773271 (PMC8894245; doi:10.3389/fpubh.2022.773271)
Supplement: Supplementary file 1 [file Data_Sheet_1.DOCX]

**Nonpharmaceutical Interventions Implemented to Control the COVID-19 were Associated with Reduction of Influenza Incidence**

**S1 Text. Classification of three time periods**

**The first period:** According to World Health Organization (WHO) (<https://covid19.who.int/region/wpro/country/sg>), the date of the first COVID-19 case was January 4, 2020, January 20, 2020, January 14, 2020, and January 23, 2020 in China, the United States, Japan, and Singapore. The time before the date of the reported COVID-19 first case was considered as the first period, when no COVID-19–specific interventions were imposed.

**The second period:** The time between the date of the first reported COVID-19 case and the date of implementing the first compulsory nonpharmaceutical intervention (S1 Table).

**The third period:** The time after the date of implementing the first compulsory nonpharmaceutical intervention, when the governments announced and implemented the most compulsory nonpharmaceutical intervention such as face mask ordinances, public gathering bans, closure of school, and closure of workplace (S1 Table).

**S1 Table.** **The dates of having a mandatory order of compulsory interventions in four countries.**

| **Compulsory interventions** | **China**^a^ | **The United States**^b^ | **Jpan** | **Singapore** |
| --- | --- | --- | --- | --- |
| Date of First Intervention | Jan. 29^th^ | Mar. 14^th^ | Feb. 25^th^ | Mar. 24^th^ |
| Face Mask Ordinances | Feb. 1^st^ | Apr. 15^th^ | Apr. 16^th^ | Apr. 16^th^ |
| Closure of School | Feb. 16^th^ | Mar. 16^th^ | Mar.2^nd^ | Apr. 8^th^ |
| Public Gathering Bans | Jan. 29^th^ | Mar. 14^th^ | Feb. 26^th^ | Mar. 24^th^ |
| Work from home | Feb. 3^rd^ | — | Feb. 25^th^ | Apr. 7^th^ |
| Visa restrictions^c^ | Mar. 28^th^ | Mar. 20^th^ | Mar. 21^th^ | Mar. 24^th^ |

^a^The first level response to public health emergencies was launched in 31 provinces nationwide in January 29, 2020 in China.

^b^Some states, including New York, Hawaii, Maryland, have had a mandatory order for everyone to wear a face mask in public since April 15, 2020 in the United States. Altogether at least 33 states have decided to close public schools, which combined with district closures in other states has shuttered least 64 000 schools, according to Education Week since March 16, 2020 in the United States. 24 states and cities have implemented bans on large gatherings on March 14, 2020.

^c^Inview of the rapid spread of COVID-19 across the world, China has decided to temporarily suspend the entry into China by foreign nationals holding visas or residence permits still valid to the time of this announcement, effective from 0 a.m., 28 march 2020. In the United States, embassies and consulates will cancel all routine immigrant and nonimmigrant visa appointments as of March 20, 2020. Visa restrictions will be implemented from 0:00 am on March 21st in Japan. Starting from 23:59 on March 23, all short-term visitors (excluding Singapore citizens, permanent residents, and long-term permit holders), regardless of nationality or origin, are not allowed to enter or transit in Singapore.

**S2 Text. Online supplement of public health documents on nonpharmaceutical interventions**

The sources of the available public health documents on nonpharmaceutical interventions for controlling COVID-19 implemented by China is available as online supplement at:

<http://www.gov.cn>

<http://paper.people.com.cn>

https://www.nia.gov.cn/n897453/c1267259/content.html

The sources of the available public health documents on nonpharmaceutical interventions for controlling COVID-19 implemented by the United States is available as online supplement at:

<https://www.cnet.com/health/where-are-face-masks-required/>

<https://www.edweek.org/ew/section/multimedia/map-coronavirus-and-school-closures.html>

<https://www.americanprogress.org/issues/healthcare/news/2020/03/14/481763/state-local-governments-must-take-much-aggressive-action-immediately-slow-spread-coronavirus/>

https://travel.state.gov/content/travel/en/News/visas-news.html

The sources of the available public health documents on nonpharmaceutical interventions for controlling COVID-19 implemented by Japan is available as online supplement at:

<https://english.kyodonews.net/news/2020/04/ffc3fc9ed997-japan-city-adopts-1st-ordinance-urging-wearing-of-masks-due-to-virus.html>

<https://www.kantei.go.jp/jp/98_abe/actions/202002/27corona.html>

<https://www.kantei.go.jp/jp/98_abe/actions/202002/26corona.html>

<https://www.mhlw.go.jp/content/10200000/000603610.pdf>

https://www.mofa.go.jp/ca/fna/page6e_000199.html

The sources of the available public health documents on nonpharmaceutical interventions for controlling COVID-19 implemented by Singapore is available as online supplement at:

<https://www.mpa.gov.sg/web/portal/home/port-of-singapore/circulars-and-notices/detail/39913650-4aee-4596-84d5-95b58bda0a33>

https://www.moe.gov.sg/news/press-releases/schools-and-institutes-of-higher-learning-to-shift-to-full-home-based-learning-preschools-and-student-care-centres-to-suspend-general-services

<https://www.mom.gov.sg/covid-19/advisory-on-safe-distancing-measures>

<https://www.moh.gov.sg/docs/librariesprovider5/pressroom/press-releases/annex-for-notification-8-apr-2020.pdf>

https://www.moh.gov.sg/news-highlights/details/additional-border-control-measures-to-reduce-further-importation-of-covid-19-cases
